# Comparative Transcriptome Analysis Reveals That WSSV IE1 Protein Plays a Crucial Role in DNA Replication Control

**DOI:** 10.3390/ijms23158176

**Published:** 2022-07-25

**Authors:** Yixi Chen, Gaochun Wu, Chuanqi Wang, Huimin Zhang, Jinghua Zhu, Yueling Zhang, Zhongyang Lin, Defu Yao

**Affiliations:** Institute of Marine Sciences and Guangdong Provincial Key Laboratory of Marine Biotechnology, Shantou University, Shantou 515063, China; 19yxchen1@stu.edu.cn (Y.C.); 17gcwu@stu.edu.cn (G.W.); chqwang@stu.edu.cn (C.W.); 20hmzhang@stu.edu.cn (H.Z.); zhujh@stu.edu.cn (J.Z.); zhangyl@stu.edu.cn (Y.Z.); linzy@stu.edu.cn (Z.L.)

**Keywords:** WSSV, shrimp, IE protein, DNA replication, MCM complex

## Abstract

For DNA viruses, the immediate-early (IE) proteins are generally essential regulators that manipulate the host machinery to support viral replication. Recently, IE1, an IE protein encoded by white spot syndrome virus (WSSV), has been demonstrated to function as a transcription factor. However, the target genes of IE1 during viral infection remain poorly understood. Here, we explored the host target genes of IE1 using RNAi coupled with transcriptome sequencing analysis. A total of 429 differentially expressed genes (DEGs) were identified from penaeid shrimp, of which 284 genes were upregulated and 145 genes were downregulated after IE1 knockdown. GO and KEGG pathway enrichment analysis revealed the identified DEGs are significantly enriched in the minichromosome maintenance (MCM) complex and DNA replication, indicating that IE1 plays a critical role in DNA replication control. In addition, it was found that *Penaeus vannamei* MCM complex genes were remarkably upregulated after WSSV infection, while RNAi-mediated knockdown of *Pv*MCM2 reduced the expression of viral genes and viral loads at the early infection stage. Finally, we demonstrated that overexpression of IE1 promoted the expression of MCM complex genes as well as cellular DNA synthesis in insect High-Five cells. Collectively, our current data suggest that the WSSV IE1 protein is a viral effector that modulates the host DNA replication machinery for viral replication.

## 1. Introduction

White spot syndrome virus (WSSV) is a large, rod-shaped, enveloped, and double-stranded circular DNA virus that belongs to the genus *Whispovirus* in the family *Nimaviridae* [1]. It infects a wide range of economic crustaceans such as shrimp, crayfish, lobster, and crab. Although WSSV has a broad host range, it exhibits the strongest pathogenicity and virulence in penaeid shrimp and causes a cumulative mortality of up to 100% within 10 days after the onset of disease in cultured shrimp [2]. First occurring in 1992, WSSV has emerged as a major pathogen that affects shrimp sustainable production worldwide. Over the past three decades, considerable progress has been made in elucidating the biology and pathogenesis of WSSV (i.e., virion genome and proteome, and viral entry and replication mechanisms) [3,4,5,6], as well as the host innate immunity against the virus (i.e., humoral and cellular immunity) [7,8]. However, there are currently no effective treatment strategies to prevent and control the rapid spread and outbreak of the viral disease. Therefore, this underscores the need for exploring the mechanisms of the WSSV–host interactions, which may contribute to new therapeutic avenues for controlling this viral disease in shrimp aquaculture.

As a DNA virus, WSSV genes are expressed following three temporal phases: immediate-early (IE), early (E), and late (L) [9]. IE genes are the first class of viral genes expressed after primary infection or reactivation of latent infection. They solely utilize the host cellular transcription and translation machinery for expression, but do not depend on any de novo synthesized viral proteins. An increasing number of studies have shown that the IE genes’ encoded products are generally regulatory proteins that initiate viral replication and/or modulate cellular functions to aid viral replication [10,11,12]. Hence, functional analysis of IE proteins is believed to be critical for understanding the virus–host interactions. Thus far, twenty-one IE genes have been identified from the WSSV genome [13,14,15]. One of WSSV IE genes, IE1, has attracted the most attention in the past decade. It has been shown that the IE1 gene has a strong promoter activity and maintains a high expression level throughout the virus life cycle [15]. The reason for this may be that IE1 could hijack various host transcription factors (e.g., STAT, NF-κB, AP-1, kruppel-like factor, YY1, etc.) to enhance its expression [16,17,18,19,20,21]. On the other hand, the biological functions of the IE1 protein have been extensively studied recently. For instance, IE1 was demonstrated to act as a transcription factor with transactivation, dimerization, and DNA-binding activity [22]. Moreover, several studies reported that IE1 could bind to many host cellular proteins such as the retinoblastoma protein (Rb) [23], STAT [19], JNK [18], β-catenin [24], Chibby [25], and prophenoloxidase (proPO) [26], thereby hijacking cellular functions or suppressing the host immunity to promote virus multiplication. The foregoing data indicate that IE1 is a multifunctional regulator that is critical for WSSV pathogenesis.

The whiteleg shrimp, *Penaeus vannamei*, is one of the most commonly farmed shrimp species around the world, which accounts for more than 80% of the global shrimp production. In the present study, we used *P. vannamei* as a model to explore the host target genes of IE1 during WSSV infection using RNAi coupled with high-throughput RNA sequencing (RNA-Seq). A total of 429 differentially expressed genes (DEGs) after IE1 knockdown were identified in WSSV-challenged shrimp. Bioinformatic analysis showed that the identified DEGs are mainly enriched in minichromosome maintenance (MCM) complex and DNA replication. In addition, we demonstrated that *P. vannamei* MCM complex genes were upregulated upon WSSV infection, while knockdown of *Pv*MCM2 suppressed the viral genes’ expression and viral replication at the early infection stage. Moreover, we found that overexpression of IE1 promoted the expression of MCM complex genes and cellular DNA synthesis in High-Five cells. Our current data reveal that IE1 is a viral effector involved in DNA replication control, which provides novel insight into the virus–host interactions.

## 2. Results

### 2.1. De Novo Assembly and Unigene Annotation

**Figure 1:**
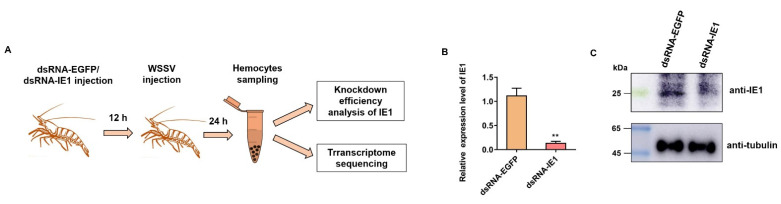
IE1 knockdown and transcriptome sequencing (**A**) Workflow of IE1 knockdown in WSSV-infected shrimp and sample preparations for transcriptome sequencing. (**B**,**C**) Knockdown efficiency analysis of IE1 using qPCR and Western blot assay. Shrimp were injected with dsRNA-IE1 and dsRNA-EGFP, respectively. At 12 h post dsRNA injection, shrimp of each group were further challenged with WSSV. After infection for 24 h, hemocytes per group were harvested and used for knockdown efficiency analysis of IE1 by qPCR and Western blot analysis, followed by transcriptome sequencing. The qPCR data were shown as mean ± SD, and the statistical significance was computed by Student’s *t*-test (** indicates *p* < 0.01).

In this study, we applied RNAi and RNA-seq techniques to explore the target genes of IE1 during WSSV infection. As shown in Figure 1A, shrimp were separately injected with the dsRNA-IE1 and the control dsRNA-EGFP, followed by WSSV challenge. The qPCR and Western blot results showed that IE1 expression significantly decreased in dsRNA-IE1-injected shrimp compared with the control dsRNA-EGFP group (Figure 1B,C), indicating a successful knockdown of IE1 during virus infection. For transcriptome sequencing, three biological replicates per group were prepared and used for RNA extraction and Illumina sequencing. As shown in Table S1, a total of 259,589,838 raw reads were generated from the above six cDNA libraries, and 254,552,182 clean reads were obtained after quality control. Next, the clean reads were mapped to *P. vannamei* genome, which led to the identification of a total of 18,652 unigenes and 24,404 transcripts, respectively. Finally, the unigenes/transcripts were searched against six functional databases, NR, Swiss-Prot, Pfam, COG, GO, and KEGG. The result showed that a total of 15,794 unigenes and 21,077 transcripts were annotated, which represented about 84.68% and 86.37% of the total, respectively (Table S2).

### 2.2. Identification and Functional Classification Analysis of DEGs

**Figure 2:**
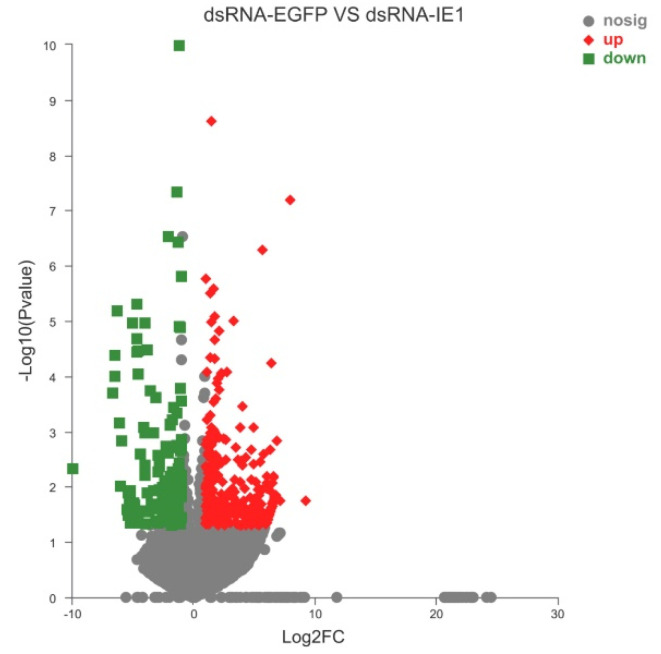
Volcano diagram of the DEGs in WSSV−infected shrimp after IE1 knockdown. The *x*−axis indicates the fold change of unigenes/transcripts between dsRNA−IE1 and dsRNA−EGFP group, and the *y*−axis shows the statistical significance of the differences (*p* value). Red dots represent the DEGs that are significantly upregulated, whereas green dots represent the DEGs that are significantly downregulated. The gray dots represent the DEGs that have no significant difference.

**Figure 3:**
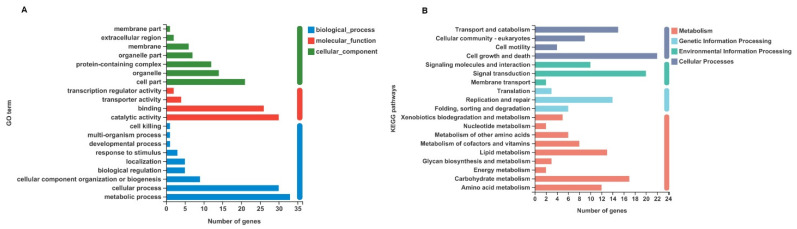
Functional classification analysis of the identified DEGs. (**A**) GO annotation analysis of the DEGs. The DEGs were classified into different GO terms according to biological process, molecular function, and cellular component. (**B**) KEGG pathway annotation analysis of the DEGs. The DEGs were categorized into various KEGG pathways according to metabolism, genetic information processing, environmental information processing, and cellular processes.

**Figure 4:**
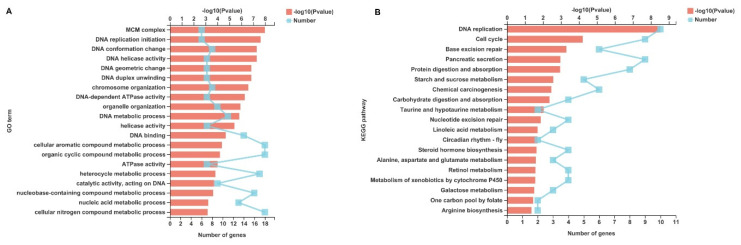
GO and KEGG pathway enrichment analysis of the identified DEGs. (**A**) Top20 of GO term enrichment. (**B**) Top20 of KEGG pathway enrichment. The *y*−axis indicates the GO term or KEGG pathway. The bottom *x*−axis indicates the number of DEGs in each GO term or KEGG pathway. The upper *x*−axis represents the significance level of enrichment.

Among the unigenes identified, unigenes with *p* < 0.05, |log2FC| ≥ 1, and fold change ≥2 or ≤0.5 were considered to be DEGs. Based on this criterion, a total of 429 DEGs were identified, of which 284 genes were upregulated (e.g., caspase8, chorion peroxidase, heme-binding protein 1, etc.) and 145 genes were downregulated (e.g., MCM2, MCM3, MCM4, MCM5, MCM7, etc.) after IE1 knockdown (Figure 2 and Table S3). To determine the biological functions of the DEGs identified, GO and KEGG pathway annotation analysis were performed. In the GO annotation analysis, the identified DEGs were classified into three categories: biological process, molecular function, and cellular component (Figure 3A). For biological process, the identified DEGs are mainly involved in the metabolic process and cellular process. In terms of cellular component and molecular function, most of the DEGs are localized in cell parts and organelles with catalytic activity and binding. In addition, the KEGG pathway annotation analysis showed that the majority of the DEGs are implicated in cell growth and death, signal transduction, and carbohydrate metabolism (Figure 3B). Furthermore, the DEGs were further subjected to GO and KEGG pathway enrichment analysis. The results showed that the most significantly enriched GO term of the DEGs was the MCM complex (Figure 4A), while DNA replication was the most significantly enriched signaling pathway (Figure 4B). Given that the MCM complex is a conserved component of the DNA replication system in all eukaryotes [27], these data indicate that IE1 might control DNA replication by modulating the MCM complex.

### 2.3. Validation of RNA-Seq Results by qPCR Analysis

**Figure 5:**
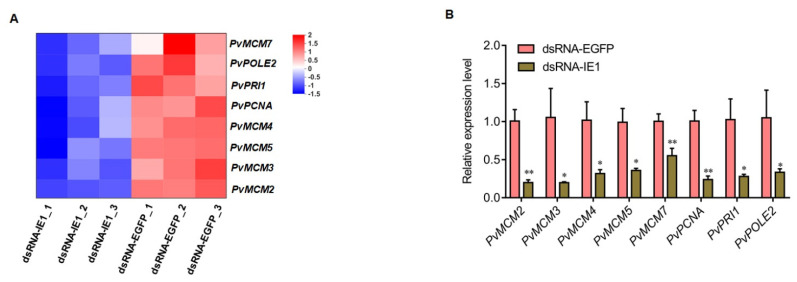
Validation of DEGs by qPCR analysis. (**A**) Heatmap of the DNA-replication-related DEGs in transcriptome sequencing data. (**B**) qPCR validation analysis of the DEGs. The data were shown as mean ± SD, and the statistical significance was determined by Student’s *t*-test (* indicates *p* < 0.05; and ** indicates *p* < 0.01).

In order to substantiate the reliability of transcriptome data, eight DEGs related to DNA replication were chosen for qPCR validation. These genes were *Pv*MCM2, *Pv*MCM3, *Pv*MCM4, *Pv*MCM5, *Pv*MCM7, proliferating cell nuclear antigen (*Pv*PCNA), DNA primase small subunit (*Pv*PRI1), and DNA polymerase epsilon subunit 2 (*Pv*POLE2). In the transcriptome sequencing analysis, these DNA replication factors were significantly downregulated in IE1-silenced shrimp compared with the control (Figure 5A). Similarly, the qPCR results confirmed that these genes also showed significant decreased expression after IE1 knockdown (Figure 5B). Therefore, the qPCR results were generally consistent with the transcriptome data, indicating the accuracy of the transcriptome data.

### 2.4. P. Vannamei MCM Complex Genes Are Upregulated after WSSV Challenge

**Figure 6:**
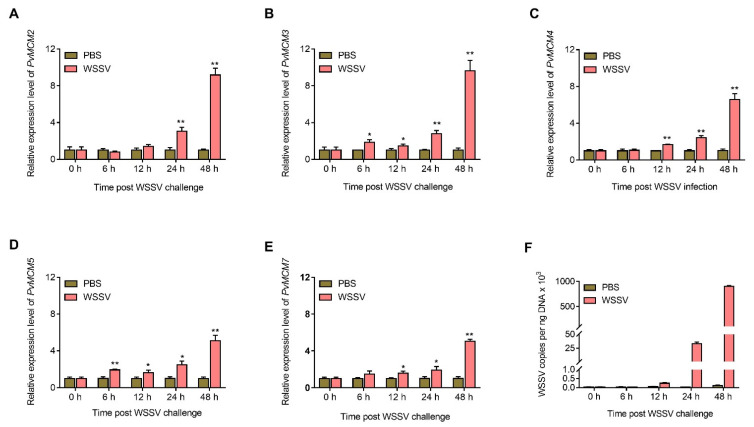
*P. vannamei* MCM complex genes are upregulated upon WSSV infection. (**A**–**E**) qPCR analysis of *Pv*MCM2, *Pv*MCM3, *Pv*MCM4, *Pv*MCM5, and *Pv*MCM7 expression after WSSV challenge. (**F**) qPCR analysis of virus copies number (viral loads). Shrimp were separately injected with WSSV or PBS (negative control). At 0, 6, 12, 24, and 48 h post viral infection, hemocytes per group were harvested and then used to determine the expression of *P. vannamei* MCM complex genes and viral loads using qPCR. All the qPCR assays were carried out in triplicate for each sample. The data were shown as mean ± SD, and the statistical significance between each group was analyzed by Student’s *t*-test (* indicates *p* < 0.05, and ** indicates *p* < 0.01).

The above results suggest that the MCM complex might have critical roles in WSSV infection. Therefore, to address this, the mRNA expression profiles of *P. vannamei* MCM complex genes (i.e., *Pv*MCM2, *Pv*MCM3, *Pv*MCM4, *Pv*MCM5, and *Pv*MCM7) in hemocytes post WSSV challenge were detected using qPCR analysis in this study. As indicated in Figure 6A–E, compared with the PBS control, the transcript levels of the analyzed MCM complex genes generally started to upregulate at 12 h, and increased sharply at 24 h and 48 h after WSSV infection. In particular, the mRNA expression of *Pv*MCM2, *Pv*MCM3, *Pv*MCM4, *Pv*MCM5, and *Pv*MCM7 was increased by approximately 9.1, 9.6, 6.5, 5.1, and 5.0 fold at 48 h post viral infection, respectively. Meanwhile, the replication of the WSSV genome during infection was evaluated by measuring the viral loads. The result showed that WSSV genome replication started at 12 h post infection, and rapidly replicated from 24 h to 48 h (Figure 6F). Thus, these results suggest that the mRNA expression of MCM complex genes is closely correlated to WSSV genome replication.

### 2.5. P. vannamei MCM Complex Enhances WSSV Replication at the Early Infection Stage

**Figure 7:**
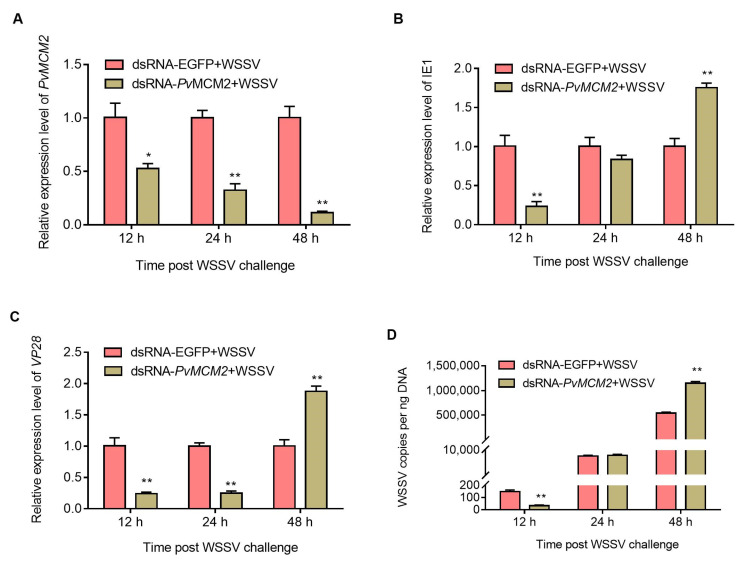
*Pv*MCM2 knockdown inhibits WSSV replication at the early infection stage. (**A**) Knockdown efficiency analysis of *Pv*MCM2. (**B**–**D**) qPCR analysis of WSSV genes’ expression (IE1 and VP28) and viral loads after *Pv*MCM2 knockdown. Shrimp were separately injected twice with dsRNA-*Pv*MCM2 and dsRNA-EGFP (negative control), followed by WSSV infection. At 12, 24, and 48 h post viral infection, hemocytes were collected and used to detect the knockdown efficiency of *Pv*MCM2, expression of WSSV genes, and viral loads using qPCR analysis. For each sample, triplicate qPCR assays were performed, and the data were shown as mean ± SD. Significant differences were computed using the Student’s *t*-test (* indicates *p* < 0.05, and ** indicates *p* < 0.01).

To further figure out the role of the *P. vannamei* MCM complex in WSSV infection, RNAi assay was performed. In this study, we selected *Pv*MCM2 for RNAi analysis as it is one of the MCM complex genes that showed the most increased expression post WSSV infection (Figure 6). First, shrimp were injected with dsRNA-*Pv*MCM2 or dsRNA-EGFP, followed by WSSV challenge. The qPCR result showed that the mRNA expression of *Pv*MCM2 in dsRNA-*Pv*MCM2-injected shrimp was reduced by about 47%, 67%, and 88% at 12, 24, and 48 h post WSSV infection, respectively, compared with the dsRNA-EGFP group (Figure 7A). This result indicated that *Pv*MCM2 was effectively silenced under WSSV infection. Subsequently, we continued to examine the mRNA expression of WSSV genes (i.e., the immediate-early gene IE1 and late gene VP28) and viral loads after *Pv*MCM2 knockdown. As shown in Figure 7B–D, compared with the dsRNA-EGFP-injected control group, the mRNA expression of IE1 and VP28 as well as viral loads in *Pv*MCM2-depleted shrimp were significantly downregulated at the early infection stage (12 h), but upregulated at the late infection period (48 h). Taken together, these data suggest that the *P. vannamei* MCM complex contributes to WSSV replication in the early stage of viral infection.

### 2.6. IE1 Promotes the Cellular DNA Synthesis In Vitro

**Figure 8:**
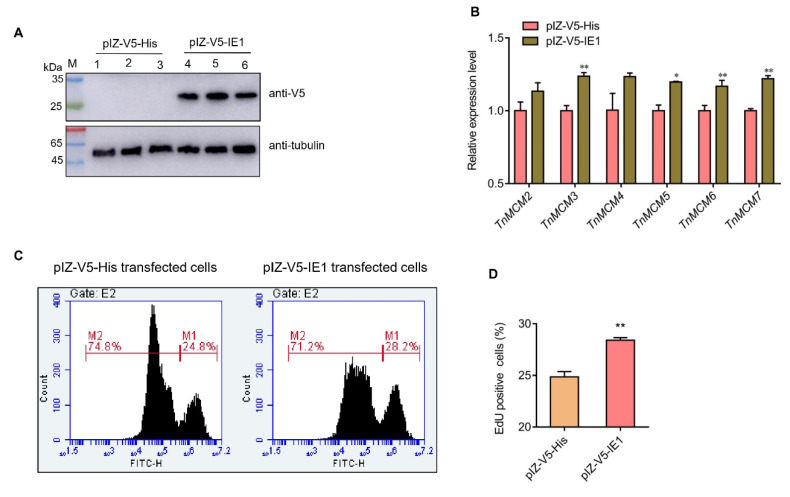
Overexpression of IE1 promotes the expression of MCM complex genes and the cellular DNA synthesis in High-Five cells. (**A**) Western blot analysis of IE1 expression in High-Five cells. (**B**) qPCR analysis of MCM complex genes’ expression after IE1 overexpression. (**C**) Analysis of DNA synthesis in IE1-expressed cells using EdU staining and flow cytometry. M2: EdU unlabeled cells, M1: EdU labeled cells. (**D**) Statistical analysis of DNA synthesis in IE1-expressed cells corresponding to (**C**). High-Five cells were transiently transfected with the plasmid pIZ-V5-IE1 and pIZ-V5-His (negative control), respectively. At 24 h post transfection, cells per group were harvested and used for Western blot/qPCR analysis and EdU staining. All the experiments were performed in triplicates, and the data were analyzed statistically by Student’s *t* test (* indicates *p* < 0.05, ** indicates *p* < 0.01).

Until now, there was no available shrimp cell line for plasmid transfections. Hence, in this study, the High-Five cell, a clonal isolate derived from the parental *Trichoplusia ni* (cabbage looper ovary), was used to determine whether IE1 could modulate DNA replication. First, we transfected the IE1 expression plasmid (pIZ-V5-IE1) and empty vector (pIZ-V5-His) into the High-Five cells and then determined the expression of MCM complex genes and DNA synthesis. As illustrated in Figure 8A, IE1 could be expressed in High-Five cells at 24 h post transfection. Following this, the pIZ-V5-IE1 and pIZ-V5-His transfected cells were harvested and used for qPCR and DNA synthesis analysis. The qPCR result showed that the mRNA expression of MCM complex genes in High-Five cells were generally upregulated after IE1 overexpression compared to the control (Figure 8B). Furthermore, the EdU-positive cells were increased by about 14% after IE1 overexpression compared with the control (Figure 8C,D). This result suggests that IE1 could facilitate the cellular DNA synthesis via elevating the expression of the MCM complex genes in vitro.

## 3. Discussion

IE1, which is an IE protein of WSSV, is a multifunctional regulator that plays critical roles in virus–host interactions. It not only functioned as a transcription factor [22], but also interacted with various shrimp cellular proteins (i.e., Rb, STAT, JNK, β-catenin, Chibby, and proPO) to manipulate the host cellular functions for viral genome replication [18,19,23,24,25,26]. In this study, RNAi coupled with transcriptome sequencing were conducted to explore the downstream genes that are regulated by IE1 directly or indirectly. Under WSSV infection, a total of 429 DEGs were identified after IE1 knockdown, including 284 upregulated genes and 145 downregulated genes (Figure 2). GO and KEGG pathway enrichment analysis revealed that the DEGs were significantly enriched in the MCM complex and DNA replication (Figure 4A,B), which suggest a role for IE1 in DNA replication control during viral infection. 

As obligate intracellular pathogens, viruses have to co-opt various host factors and machineries to replicate their genomes. Thus far, although WSSV encodes several DNA replication factors such as DNA polymerase, helicase, and dUTPase [3,28,29], it is still unclear how WSSV replicates its genome and what are key regulators that are involved in initiating genome replication. Here, our transcriptome sequencing and qPCR results showed that IE1 depletion could inhibit the expression of many DNA replication factors (especially MCM complex genes) under WSSV invasion (Figure 5A,B). In eukaryotic cells, the MCM complex is a hetero–hexameric protein complex composed of MCM2-MCM7. It is thought to function as a DNA replicative helicase that binds to the replication origin and unwinds double-stranded DNA (dsDNA) to initiate replication. Recently, there is growing evidence that many viruses hijack the cellular MCM complex to support viral genome replication. For example, Chaudhuri et al. have shown that Epstein-Barr virus (EBV) recruits the MCM proteins for the propagation of its genome [30]. Kawaguchi et al. have demonstrated that the interaction of MCM proteins and the PA subunit of the viral RNA-dependent RNA polymerase is required for de novo replication of the influenza virus RNA genome [31]. Recently, Dabral et al. reported that Kaposi’s sarcoma-associated herpesvirus (KSHV)-encoded latency-associated nuclear antigen (LANA) interacted with the host MCM proteins (i.e., MCM3, MCM4, and MCM6) and recruited them to the viral replication origin for viral DNA replication [32]. Intriguingly, our present studies showed that the mRNA expression of *P. vannamei* MCM complex genes was found to be upregulated in response to WSSV attack (Figure 6), while RNAi-mediated suppression of *Pv*MCM2 downregulated the expression of WSSV genes and viral loads at the early infection stage (12 h) (Figure 7). Furthermore, we demonstrated that overexpression of IE1 could enhance the expression of MCM complex genes and cellular DNA synthesis in High-Five cells (Figure 8). Based on the above findings, we propose that IE1 can modulate the cellular DNA replication machinery via targeting the MCM complex, which ultimately promotes the replication of the WSSV genome.

It is worth mentioning that in contrast to the early infection stage (i.e., 12 h), knockdown of *Pv*MCM2 increased the WSSV genes’ expression and viral loads at the late infection period (i.e., 48 h) (Figure 7), indicating that the MCM complex may play important roles in the host defensive immunity. Consistent with this result, several studies recently have reported that the MCM complex protein from shrimp and crab could participate in the antiviral and/or antibacterial immune response by regulating phagocytosis, apoptosis, and expression of immune genes [33,34]. Given that the MCM complex may play critical roles in immune defense, coupled with the fact that WSSV can encode its own helicase [3], we speculated that WSSV may utilize the host cellular helicase (i.e., MCM complex) to initiate its genome replication at the early phase of infection, but depends on its own helicase at the late stage. Consequently, the MCM complex could be required for the initial genome replication of WSSV, whilst plays antiviral roles at the late infection stage. In addition, although our results revealed that IE1 could modulate the expression of MCM complex genes during WSSV infection (Figure 5), the molecular mechanisms behind this remain unclear. Interestingly, given that IE1 not only functions as a transcription factor by itself, but also interacts with the host transcription factors (e.g., STAT and TATA-box-binding protein) [19,22,35], it is necessary for us to explore whether IE1 directly modulates the expression of the host MCM complex genes in the future. On the other hand, it has been previously shown that expression of MCM complex genes could be regulated by the cell cycle transcription factor E2F [36,37]. A recent work suggests that IE1 can interact with the host Rb protein, thereby resulting in the release and activation of E2F [23]. These data hint that IE1 may modulate the expression of MCM proteins via the Rb-E2F pathway.

In conclusion, this study for the first time explored the downstream genes of IE1 during WSSV infection using RNAi and transcriptome sequencing analysis. A total of 429 DEGs after IE1 knockdown were identified, which are mainly involved in DNA replication. Our results reveal that IE1 could modulate the cellular DNA replication machinery via regulating the MCM complex and consequently drives viral genome replication. The current data provide a novel insight into the biological functions of IE1, which contributes to better understanding of WSSV–host interactions and therefore helps in developing more potential therapeutic avenues for virus infection.

## 4. Materials and Methods

### 4.1. Shrimp Culture and Virus

The penaeid shrimp, *P. vannamei* (as 5–8 g body weight), was purchased from Huaxun Aquatic Product Corporation (Shantou, China). These shrimp were acclimatized for 2 days in tanks with aerated seawater at room temperature before experiments. The WSSV strain used in this study was the Chinese mainland isolate (GenBank accession no: AF332093). The virus inoculum was isolated from WSSV-infected crayfish (Procambrus clarkii) and quantified as previously described [38,39]. The animal experiment was reviewed and approved by the Animal Research and Ethics Committees of Shantou University, Guangdong, China.

### 4.2. RNAi-Mediated Knockdown of IE1 and Sample Collection

The gene silencing of IE1 during viral infection was carried out according to a previous protocol with minor modifications [35]. Briefly, the double-stranded RNA (dsRNA) targeting the IE1 gene (dsRNA-IE1) was prepared in vitro using the T7 RiboMAX^TM^ Express RNAi System (Promega, Madison, WI, USA). The dsRNA specific for the EGFP gene (dsRNA-EGFP) was also synthesized for a negative control. The primers used for dsRNA synthesis are shown in Table S4. For RNAi assay, shrimp were randomly divided into two groups. The experimental group was intramuscularly injected with 10 µg of dsRNA-IE1, while the control group was injected with an equal amount of dsRNA-EGFP. At 12 h post dsRNA injection, each shrimp per group was further challenged with 100 µL of WSSV virions (1 × 10^5^ copies). At 24 h after viral infection, the hemolymph of each group was collected from the pericardial sinus using a sterile syringe and immediately mixed with ice-cold anti-coagulant solution (258 mM sodium citrate dihydrate, 328 mM sodium citrate, 110 mM glucose, 140 mM NaCl, pH 6.0). The hemocytes were harvested by centrifugation at 500 g for 10 min at 4 °C, and then used for RNA extraction and protein sample preparation. Before transcriptome sequencing, the IE1 knockdown efficiency was evaluated using qPCR and Western blot analysis as described in Section 4.6 and Section 4.10. The qPCR primers of IE1 and the internal reference gene *Pv*EF1α are shown in Table S4. The primary antibody against IE1 (anti-IE1) was previously prepared by immunizing the mouse with purified recombinant IE1 in our lab.

### 4.3. RNA Extraction, cDNA Library Construction, and Transcriptome Sequencing

Total RNA was extracted from the hemocytes of each group using an RNAfast200 kit (Fastagen, Shanghai, China) according to the manufacturer’s instructions, and genomic DNA was removed using DNase I (Takara bio Inc., Dalian, China). Then, RNA quality was determined by the Agilent 2100 Bioanalyser, while RNA concentration was quantified using the NanoDrop 2000 spectrophotometer. Only high-quality RNA samples (OD260/280 = 1.8~2.2, OD260/230 ≥ 2.0, RIN ≥ 6.5) were used to construct cDNA libraries. To prepare cDNA libraries for sequencing, mRNA with poly (A) tail was isolated from the total RNA using oligo(dT) magnetic beads and then randomly fragmented into ~300 bp fragments. Thereafter, the double-stranded cDNA was synthesized using a SuperScript double-stranded cDNA synthesis kit (Invitrogen, Waltham, MA, USA) with random hexamer primers. The synthesized cDNA was then subjected to end-repair, phosphorylation, ‘A’ base addition, and adapter connection. Libraries were size selected for cDNA target fragments of 300 bp on 2% low-range ultra-agarose, followed by PCR amplified using Phusion DNA polymerase (NEB) for 15 PCR cycles. After purification and quantification, the paired-end cDNA libraries were sequenced with the Illumina NovaSeq 6000 platform at Shanghai Majorbio Bio-pharm Technology Co., Ltd. (Shanghai, China).

### 4.4. Reads Mapping, De Novo Assembly, and Unigene Annotation

The generated raw reads of each sample were trimmed for quality control using fastp program (https://github.com/OpenGene/fastp). Next, the clean reads were separately aligned to the reference genome of Penaeus vannamei (GenBank accession no: ASM378908v1) using HISAT2 software (https://daehwankimlab.github.io/hisat2/), and the mapped reads were then subjected to de novo assembly using StringTie software (http://ccb.jhu.edu/software/stringtie/). Finally, the obtained unigenes/transcripts were searched against six functional databases: NCBI non-redundant protein (NR), Swiss-Prot, Pfam, Clusters of Orthologous Groups of Proteins (COG), Gene Ontology (GO), and Kyoto Encyclopedia of Genes and Genomes (KEGG), respectively.

### 4.5. Identification and Functional Classification Analysis of Differential Expression Genes

The transcript abundance of unigenes/transcripts was calculated by RSEM software (http://deweylab.biostat.wisc.edu/rsem/) according to the transcripts per million reads (TPM) value. The differential expression genes (DEGs) after IE1 knockdown were identified using the DESeq2 software [40]. In addition, GO and KEGG pathway enrichment analysis of DEGs were carried out using GOATOOLS (https://github.com/tanghaibao/goatools) and KOBAS (http://kobas.cbi.pku.edu.cn).

### 4.6. Validation of DEGs by Real-Time Quantitative PCR (qPCR) Analysis

To validate the transcriptome data, total RNA was separately extracted from the hemocytes of dsRNA-IE1 and dsRNA-EGFP group, and then reverse-transcribed into cDNA using TransScript one-step gDNA removal and cDNA synthesis superMix (TransGen Biotech, Beijing, China) following the manufacturer’s instructions. Eight DEGs associated with DNA replication were selected for qPCR analysis using a LightCycler 480 system (Roche, Basel, Switzerland). Each qPCR reaction mixture included 10 μL of 2×RealStar Green power mixture (GenStar, Beijing, China), 1 μL of each forward and reverse primer (10 μM), 1 μL of cDNA, and 7 μL of ddH2O. The qPCR reactions were performed under the following cycling conditions: 1 cycle at 95 °C for 5 min, followed by 40 cycles at 95 °C for 15 s and 60 °C for 30 s. Each sample per group was carried out in triplicate, and the relative change in gene expression was calculated using the 2^−ΔΔCT^ method [41] and normalized to the internal control gene *Pv*EF1α. The primers of DEGs used for qPCR analysis are shown in Table S4. 

### 4.7. Expression Analysis of P. vannamei MCM Complex Genes after WSSV Infection

For the WSSV challenge experiment, shrimp were intramuscularly injected with 100 µL of WSSV virions (1 × 10^5^ copies) using a sterile syringe. Meanwhile, an equivalent volume of sterile PBS was injected into shrimp and used as a negative control. At 0, 6, 12, 24 and 48 h post infection, hemocytes of each group were collected from four individual shrimp and then used for RNA and DNA isolation. The isolated RNA was then subjected to cDNA synthesis to determine the relative mRNA expression of *P. vannamei* MCM complex genes by qPCR analysis, as depicted above. The primer sequences of *P. vannamei* MCM complex genes are listed in Table S4. In addition, in order to assess whether WSSV is well-propagated, the genomic DNA per group was extracted using the TIANamp Marine Animals DNA Kit (TianGen, Beijing, China) and used to calculate the viral loads using absolute qPCR according to our previous method [42]. 

### 4.8. Detection of Viral Genes’ Expression and Viral Loads after PvMCM2 Knockdown

To explore the roles of the *P. vannamei* MCM complex in WSSV infection, RNAi assay was performed as described above with slight modification. Briefly, the dsRNA specific for *Pv*MCM2 and EGFP (designated as dsRNA-*Pv*MCM2 and dsRNA-EGFP) was synthesized in vitro using a commercial dsRNA synthesis kit. The primer sequences used for dsRNA synthesis are provided in Table S4. For the RNAi experiment, shrimp were intramuscularly injected with 10 μg of dsRNA-*Pv*MCM2 or dsRNA-EGFP (negative control) using a sterile syringe with a 22-gauge needle, followed by another injection with 5 μg of dsRNA-*Pv*MCM2 or dsRNA-EGFP after 24 h. Next, shrimp from each group were further injected with 100 μL of WSSV virions (1 × 10^5^ copies) at 24 h post the second dsRNA injection. At 12, 24, and 48 h post virus infection, hemocytes per group were harvested and immediately subjected to isolate the RNA and DNA. Finally, after evaluating the knockdown efficiency of *Pv*MCM2, the mRNA expression of WSSV genes (i.e., IE1 and VP28) and viral loads were quantified using qPCR analysis as described above. 

### 4.9. Plasmid Transfection and Analysis of MCM Complex Genes’ Expression and DNA Synthesis

High-Five (BTI-TN-5B1-4) cells were seeded on a 24-well cell culture plate and maintained in Express Five SFM medium (Invitrogen, Waltham, MA, USA) overnight. For plasmid transfection, the IE1 expression plasmid pIZ-V5-IE1 (1 µg), which was constructed in our previous study [26], was transfected into cells using the FuGENE HD transfection reagent (Promega, Madison, WI, USA) following manufacturer’s instructions. An equal amount of empty vector pIZ-V5-His (Invitrogen, Waltham, MA, USA) was transfected and used as a negative control. At 24 h post transfection, cells were harvested and used for qPCR and DNA synthesis analysis. The qPCR assays were performed as described in Section 4.6, and the primers used for amplification of MCM complex genes and internal reference (EF1α) are shown in Table S4. The DNA synthesis assays were carried out using BeyoClick™ EdU Cell Proliferation Kit with Alexa Fluor 488 (Beyotime, Nantong, China) according to manufacturer’s protocol. In brief, the 5-Ethynyl-20-deoxyuridine (EdU) agent was added to each well and incubated with cells for 3 h. The cells were then fixed in 4% paraformaldehyde (PFA) for 20 min at room temperature. After three washes with PBS containing 3% BSA, the cells were permeabilized with PBS containing 0.3% TritonX-100 for 10 min, followed by three washes with PBS containing 3% BSA. Lastly, the cells were incubated with Click Reaction System containing Alexa Fluor 488 for 30 min at room temperature. After three washes with PBS, a flow cytometer and BD Accuri™ C6 Plus software (BD Biosciences, Franklin Lakes, NJ, USA) were used for EdU detection.

### 4.10. Western Blot Analysis

The High-Five cells transfected with the plasmid pIZ-V5-His or pIZ-V5-IE1 were harvested and lysed using Western and IP cell lysis buffer (Beyotime, Nantong, China). The prepared protein samples were separated by SDS-PAGE and then electroblotted onto PVDF membranes (Millipore, Burlington, MA, USA). Next, the membranes were blocked with 5% skim milk dissolved in TBST (20 mM Tris, 150 mM NaCl, 0.1% Tween 20, pH 7.6) at room temperature for 1 h. After blocking, the membranes were incubated with mouse anti-V5 (Sangon, Shanghai, China) or anti-tubulin (Sigma-Aldrich, St. Louis, MO, USA) for 2 h at room temperature, followed by incubation with the HRP-conjugated goat anti-mouse IgG secondary antibody (Invitrogen, Waltham, MA, USA) for 1 h at room temperature. After three washes, the membranes were then reacted with Immobilon Western Chemiluminescent HRP Substrate (Millipore, Burlington, MA, USA), and protein signals were detected using Amersham Imager 600 (GE Healthcare, Chicago, IL, USA).

## Data Availability

The transcriptome sequencing data have been deposited to the GenBank database under accession number PRJNA827974. The data supporting the findings of this study are available within the article and its Supplementary Materials.

## Supplementary Materials

The following supporting information can be downloaded at: https://www.mdpi.com/article/10.3390/ijms23158176/s1.
