# Supplementary material for: Comparative Transcriptome Analysis Reveals That WSSV IE1 Protein Plays a Crucial Role in DNA Replication Control
Source: Int J Mol Sci. 2022 Jul 25;23(15):8176. doi: 10.3390/ijms23158176 (PMC9330391; doi:10.3390/ijms23158176)
Supplement: Supplementary file 1 [file ijms-23-08176-s001.zip › Table S4.pdf]

Table S4 The primer sequences used in this study

Primer name	Sequence (5’-3’)
**For qPCR assay**	
qIE1-F	GCACAACAACAGACCCTACCC
qIE1-R	GAAATACGACATAGCACCTCCAC
qVP28-F	AAACCTCCGCATTCCTGTGA
qVP28-F	TCCGCATCTTCTTCCTTCAT
q*Pv*MCM2-F	GGCGAGACCAAGAACCCC
q*Pv*MCM2-R	GCCGTCAAACCAACAGCG
q*Pv*MCM3-F	TGGTCAGCTGCCTCGCTC
q*Pv*MCM3-R	GAACCCTCCCTGCTTATTGG
q*Pv*MCM4-F	TCCCAGAGATGCGAGAGGC
q*Pv*MCM4-R	TGAGAGCGATTGTGAATGAGAGTA
q*Pv*MCM5-F	CTTTGTGATGGAGGGTGGAG
q*Pv*MCM5-R	CCTTGGCAATGGAGATGGT
q*Pv*MCM7-F	ATTCGCAACAGATACCCACCA
q*Pv*MCM7-R	CATAGGCTTCACTTCCGTACACC
q*Pv*PCNA-F	AGGTTTCACTTTCTATGTCCCCT
q*Pv*PCNA-R	TCGTCCTCAATCTTGGGGG
q*Pv*PRI1-F	TCTTATGGGTCTATTCTGGTCGTC
q*Pv*PRI1-R	CTTCTGTGTGATGTTAACTTTCTTGC
q*Pv*POLE2-F	TCCCTCCTCCTGAAACACCA
q*Pv*POLE2-R	AACCATACATCTGACATCACCACA
q*Pv*EF1α-F	TATGCTCCTTTTGGACGTTTTGC
q*Pv*EF1α-R	CCTTTTCTGCGGCCTTGGTAG
q*Tn*MCM2-F	CGGTCTTACTGCCTATGTG
q*Tn*MCM2-R	CCTGGTCGTTCATCTTGTC
q*Tn*MCM3-F	CGACCTGCTGTTCGTGAT
q*Tn*MCM3-R	CGATTGGGTTGTTTCCTC
q*Tn*MCM4-F	TCACAGGATGACGAGGAT
q*Tn*MCM4-R	ATGAGCGACTCCAACTGA
q*Tn*MCM5-F	TCAGGCAAAGGTTCCAGT
q*Tn*MCM5-R	CAACTCTGTCGTCCTCCC
q*Tn*MCM6-F	GGCGACTCGGCACCTAAA
q*Tn*MCM6-R	TCTGTCCTTGTCCCACCA
q*Tn*MCM7-F	CGCCATCCTGCGTCTATC
q*Tn*MCM7-R	GCGGTCCGTCGAGCTTAT
q*Tn*EF1α-F	CATTTCTCGTCCATTCTGTT
q*Tn*EF1α-R	CCACCGCATTTGTAGATC
**For RNAi assay**	
dsRNA-IE1-F	GAAGACTCTACAAATCTCTTT
dsRNA-IE1-R	CTTGCACCTACACGCATTACA
dsRNA-IE1-T7-F	GGATCCTAATACGACTCACTATAGGGAAGACTCTACAAATCTCTTT

dsRNA-IE1-T7-R	GGATCCTAATACGACTCACTATAGGCTTGCACCTACACGCATTACA
dsRNA-EGFP-F	CGTAAACGGCCACAAGTT
dsRNA-*Pv*MCM2-F	GCCCATTTGTCCAGTCTCA
dsRNA-*Pv*MCM2-R	CATCTTTGCTCAGGGATACG
dsRNA-*Pv*MCM2-T7-F	GGATCCTAATACGACTCACTATAGGGCCCATTTGTCCAGTCTCA
dsRNA-*Pv*MCM2-T7-R	GGATCCTAATACGACTCACTATAGGCATCTTTGCTCAGGGATACG
dsRNA-EGFP-F	TTCACCTTGATGCCGTTC
dsRNA-EGFP-T7-F	GGATCCTAATACGACTCACTATAGGCGTAAACGGCCACAAGTT
dsRNA-EGPF-T7-R	GGATCCTAATACGACTCACTATAGGTTCACCTTGATGCCGTTC
